# The effect of prednisolone and a short-term prednisolone discontinuation for the diagnostic accuracy of FDG-PET/CT in polymyalgia rheumatica—a prospective study of 101 patients

**DOI:** 10.1007/s00259-024-06697-8

**Published:** 2024-04-02

**Authors:** Andreas Wiggers Nielsen, Ib Tønder Hansen, Berit Dalsgaard Nielsen, Søren Geill Kjær, Jesper Blegvad-Nissen, Kate Rewers, Christian Møller Sørensen, Ellen-Margrethe Hauge, Lars Christian Gormsen, Kresten Krarup Keller

**Affiliations:** 1 Department of Rheumatology, Aarhus University Hospital, Led- Og Bindevævssygdomme, Palle Juul-Jensens Boulevard 59, 8200 Aarhus, Denmark; 2 Department of Clinical Medicine, Aarhus University, Aarhus, Denmark; 3 Diagnostic Centre, Silkeborg Regional Hospital, Silkeborg, Denmark; 4 Department of Internal Medicine, Horsens Regional Hospital, Horsens, Denmark; 5 Department of Nuclear Medicine and PET, Odense University Hospital, Odense, Denmark; 6 Department of Nuclear Medicine and PET, Aarhus University Hospital, Aarhus, Denmark

**Keywords:** Polymyalgia rheumatica, FDG-PET/CT, Diagnostic imaging, Effect of prednisolone

## Abstract

**Purpose:**

2-[18F]Fluoro-2-deoxy-D-glucose (FDG)–positron emission tomography (PET)/computed tomography (CT) has been suggested as an imaging modality to diagnose polymyalgia rheumatica (PMR). However, the applicability of FDG-PET/CT remains unclear, especially following glucocorticoid administration. This study aimed to investigate the diagnostic accuracy of FDG-PET/CT before and during prednisolone treatment, as well as following short-term prednisolone discontinuation.

**Methods:**

Treatment naïve suspected PMR patients were clinically diagnosed at baseline and subsequently had an FDG-PET/CT performed. Patients diagnosed with PMR were administered prednisolone following the first FDG-PET/CT and had a second FDG-PET/CT performed after 8 weeks of treatment. Subsequently, prednisolone was tapered with short-term discontinuation at week 9 followed by a third FDG-PET/CT at week 10. An FDG-PET/CT classification of PMR/non-PMR was applied, utilizing both the validated Leuven score and a dichotomous PMR score. The final diagnosis was based on clinical follow-up after 1 year.

**Results:**

A total of 68 and 27 patients received a final clinical diagnosis of PMR or non-PMR. A baseline FDG-PET/CT classified the patients as having PMR with a sensitivity/specificity of 86%/63% (Leuven score) and 82%/70% (dichotomous score). Comparing the subgroup of non-PMR with inflammatory diseases to the PMR group demonstrated a specificity of 39%/54% (Leuven/dichotomous score). After 8 weeks of prednisolone treatment, the sensitivity of FDG-PET/CT decreased to 36%/41% (Leuven/dichotomous score), while a short-term prednisolone discontinuation increased the sensitivity to 66%/60%.

**Conclusion:**

FDG-PET/CT has limited diagnostic accuracy for differentiating PMR from other inflammatory diseases. If FDG-PET/CT is intended for diagnostic purposes, prednisolone should be discontinued to enhance diagnostic accuracy.

**Trial registration:**

ClinicalTrials.gov (NCT04519580). Registered 17th of August 2020.

**Supplementary information:**

The online version contains supplementary material available at 10.1007/s00259-024-06697-8.

## Introduction

Diagnosing polymyalgia rheumatica (PMR) can be difficult, and it has been reported that 10–30% of patients are misclassified by rheumatologists at the initial evaluation [1–3]. To address this issue, several classification criteria have been developed, but neither these nor a clinical diagnosis alone have exhibited a high diagnostic performance [4–10]. As a result, specific diagnostic tests are currently unavailable, and an accurate diagnosis still relies on clinical follow-up [11–13]. Another common clinical issue is that 50% of patients with suspected PMR are administered prednisolone prior to rheumatologic assessment [14]. This can mask the symptoms of PMR as well as important differential diagnoses. Consequently, patients may require a prednisolone taper to confirm or reject the PMR diagnosis [15–17].

2-[18F]Fluoro-2-deoxy-D-glucose (FDG)–positron emission tomography (PET)/computed tomography (CT) has recently been suggested as a potential imaging modality for diagnosing PMR [11, 12]. It has been argued that a characteristic FDG-uptake pattern at specific musculoskeletal sites may be pathognomonic for PMR [18]. Several FDG-PET/CT scores and algorithms have been developed, which assess FDG uptake in the most characteristic sites around the shoulders, hips, and spine [11, 12, 19–24]. In particular, the Leuven composite score has shown promising results with a sensitivity of 85% and specificity of 88% for the PMR diagnosis, which has recently been validated in two additional studies [11, 12, 25]. However, in all three studies, the control groups primarily comprised patients without inflammatory diseases. Such patients may not demonstrate increased musculoskeletal FDG uptake due to their state of limited inflammation, resulting in inflated diagnostic performances of FDG-PET/CT. Furthermore, only the original study by Henckaerts et al. had a prospective design, whereas the validation studies were performed on retrospective cohorts. Consequently, there is a need for prospective studies including individuals with other inflammatory diseases to ensure a comprehensive evaluation. Moreover, the applicability of FDG-PET/CT for diagnosing patients with PMR after the commencement of prednisolone needs clarification, particularly whether patients should be discontinued in prednisolone prior to the FDG-PET/CT scan.

The aim of this study was to investigate the diagnostic accuracy of FDG-PET/CT in patients suspected of PMR and further to evaluate the effects of prednisolone treatment and short-term prednisolone discontinuation.

## Material and methods

### Patient population and design

Consecutive patients suspected of PMR were prospectively included between September 2020 and June 2022 from the Departments of Rheumatology at Aarhus University Hospital, Horsens Regional Hospital, and Silkeborg Regional Hospital in Denmark. To assess all potential patients with PMR, rapid referral clinics were established for all individuals suspected of PMR, and general practitioners were informed about this new service through information meetings and advertisements in newsletters dedicated to general practitioners. After referral, all patients were evaluated in the clinics preferably within 1 week. Inclusion criteria for the study were as follows: patients suspected of PMR, aged over 50, and experiencing proximal muscle pain. The exclusion criteria were as follows: any glucocorticoid treatment within the last 3 months; a medical history of PMR or giant cell arteritis (GCA); any other inflammatory rheumatic disease; symptoms of cranial GCA including headache, jaw claudication, scalp tenderness, or sight disturbances; and any active malignant cancers within the past 5 years (except basal cell carcinoma [26]).

The study design is graphically depicted in Supplementary Figure S1. At baseline, all patients had their medical history taken, lab work; were clinically examined, had vascular ultrasound as well as ultrasound of shoulders and hips performed; and were initially clinically diagnosed by the treating clinician. Subsequently, all patients had an FDG-PET/CT performed within days and before initiation of prednisolone treatment. Patients diagnosed with GCA subsequently to the FDG-PET/CT were excluded from the final analyses. Patients with a clinical diagnosis of solely PMR were administered 15 mg of prednisolone with a gradual taper (Supplementary Figure S1). If an adequate treatment response was not achieved within 2–4 weeks, based on lab work and telephone consultations at week 1 and week 4, the prednisolone dose was increased to 25 mg daily. Additionally, patients experiencing incomplete remission at week 1 underwent an additional telephone consultation along with lab work to assess whether an early increase in prednisolone dosage at week 2 was indicated. After 8 weeks of prednisolone treatment, a second clinical visit was performed consisting of a clinical examination, lab work, and a second FDG-PET/CT. Patients in clinical remission at week 8 were tapered to 5 mg prednisolone for 1 week followed by prednisolone discontinuation for a week. At week 10, during the temporary treatment discontinuation, a similar clinical visit was conducted including a third FDG-PET/CT. Prednisolone was subsequently restarted and tapered according to standard care [27]. Patients with incomplete remission at week 8 or relapse during prednisolone discontinuation continued or restarted prednisolone administration and were withdrawn from the week 10 visit, since this visit aimed at evaluating the effect of a short-term prednisolone discontinuation. At the 1-year follow-up, an experienced rheumatologist (KKK, ITH, BDN, CMS, SGK, JBN) determined the final diagnosis, including patients withdrawn from previous visits, considering the initial baseline diagnosis and the progression of the disease during the first year. This 1-year diagnosis served as the reference standard in this study. Patients initially diagnosed with other conditions, GCA or cancer, at the baseline visit received a telephone consultation and a medical chart review after 1 year to confirm their alternative diagnosis.

The study has been registered at the ClinicalTrials.gov database 17th of August 2020 (NCT04519580). Study data was collected and managed using REDCap electronic data capture tools hosted at Aarhus University [28, 29]

### PMR activity

At each visit, the clinician independently assessed whether PMR patients were in remission or relapsed based on their clinical expertise, since a widely accepted scoring system for relapse is lacking. The PMR activity score (PMR-AS) was assessed: PMR-AS score < 7 indicating low PMR activity, 7–17 representing medium disease activity, and a score of more than 17 indicating high disease activity [30].

### Blood samples

At baseline, laboratory tests were performed to investigate potential differential diagnoses. These tests included assessment of creatine kinase; p-25-hydroxy vitamin D2 + D3; thyroid-stimulating hormone; Ca2 + ; M-component; kappa and lambda chains; immunoglobulin A, G, and M; rheumatoid factor; anti-citrullinated-protein-antibody; and erythrocyte sedimentation rate (ESR). Routine tests were conducted at each visit including C-reactive protein (CRP), creatinine, alanine aminotransferase, alkaline phosphatase, platelet count, hemoglobin, white blood cell count, absolute neutrophil count, and absolute lymphocyte count.

### FDG-PET/CT scan procedure

Before the FDG-PET/CT scans, patients underwent a minimum fasting period of 6 h, except for diabetic patients who fasted for a minimum of 4 h. Patients received an intravenous infusion of 4 MBq FDG per kilogram body weight 60 min before undergoing a scan from the vertex of the skull to the mid-thigh using an integrated PET/CT scanner with continuous bed motion (Siemens Biograph Vision). Images were reconstructed using 4 iterations, 5 subsets, and 2-mm Gaussian post-processing filter, in matrix size 440 × 440. During the study, the FDG-PET/CT protocol was updated, and patients were subsequently instructed to have their arms down during the scan, aligning with the latest recommendation [31, 32]. An initial low-dose CT was performed for attenuation correction and anatomic mapping.

### FDG-PET/CT evaluation

Two experienced nuclear medicine specialists (LCG and KR) and a trained medical doctor (AWN) independently evaluated the baseline, 8-week, and 10-week FDG-PET/CT scans blinded from the clinical diagnosis, clinical data, and scanning date. In accordance with standard daily procedures for FDG-PET/CT evaluation, each evaluator initially provided a binary classification of PMR (PMR yes/no) for each patient based on their regular practice, and any discrepancies were resolved through discussion between AWN, LCG, and KR. Furthermore, 12 different anatomic sites were evaluated for FDG uptake: shoulder joints, sternoclavicular joints, ischial tuberosities, hip joints, greater trochanters, and cervical/lumbar interspinous bursae. The FDG uptake was visually graded for each site and compared to liver uptake; 0, no uptake; 1, uptake lower than liver; 2, uptake equal to or higher than liver [11]. A summed PET-PMR score was calculated for each patient using the Leuven score and a cut-off of 16 or above was considered positive for PMR [11].

### Sample size

Based on the only other prospective FDG-PET study in this research area, approximately 99 patients should be enrolled to obtain 95% confidence interval (95% CI) at baseline for a sensitivity of 85% (95% CI, 76–91) and a 95% CI for a specificity of 87% (95% CI, 79–93) [11].

### Statistics

Clinical and FDG-PET/CT data between groups were compared with Student’s *t*-test or Mann–Whitney *U* test for continuous variables and chi-square statistics or a Fisher’s exact test for categorical variables. Sensitivity and specificity were calculated for a positive FDG-PET/CT using the Leuven score as well as the dichotomous diagnostic score. The interrater variability of the Leuven score between the three FDG-PET/CT raters was assessed with intraclass correlation coefficient (ICC) based on a mean-rating, absolute-agreement, two-way mixed-effects model [33]. An ICC estimate within the range of < 0.50, 0.50–0.75, 0.75–0.90, and > 0.90 corresponds to reliability levels categorized as poor, moderate, good, and excellent, respectively [34]. Repeated measurements within the same individuals were assessed using repeated measures ANOVA followed by a Bonferroni post hoc test. Statistical analyses were performed using STATA (version 17, StataCorp, USA). Two-tailed *p*-values less than 0.05 were considered statistically significant.

## Results

### Patient characteristics

**Fig. 1:**
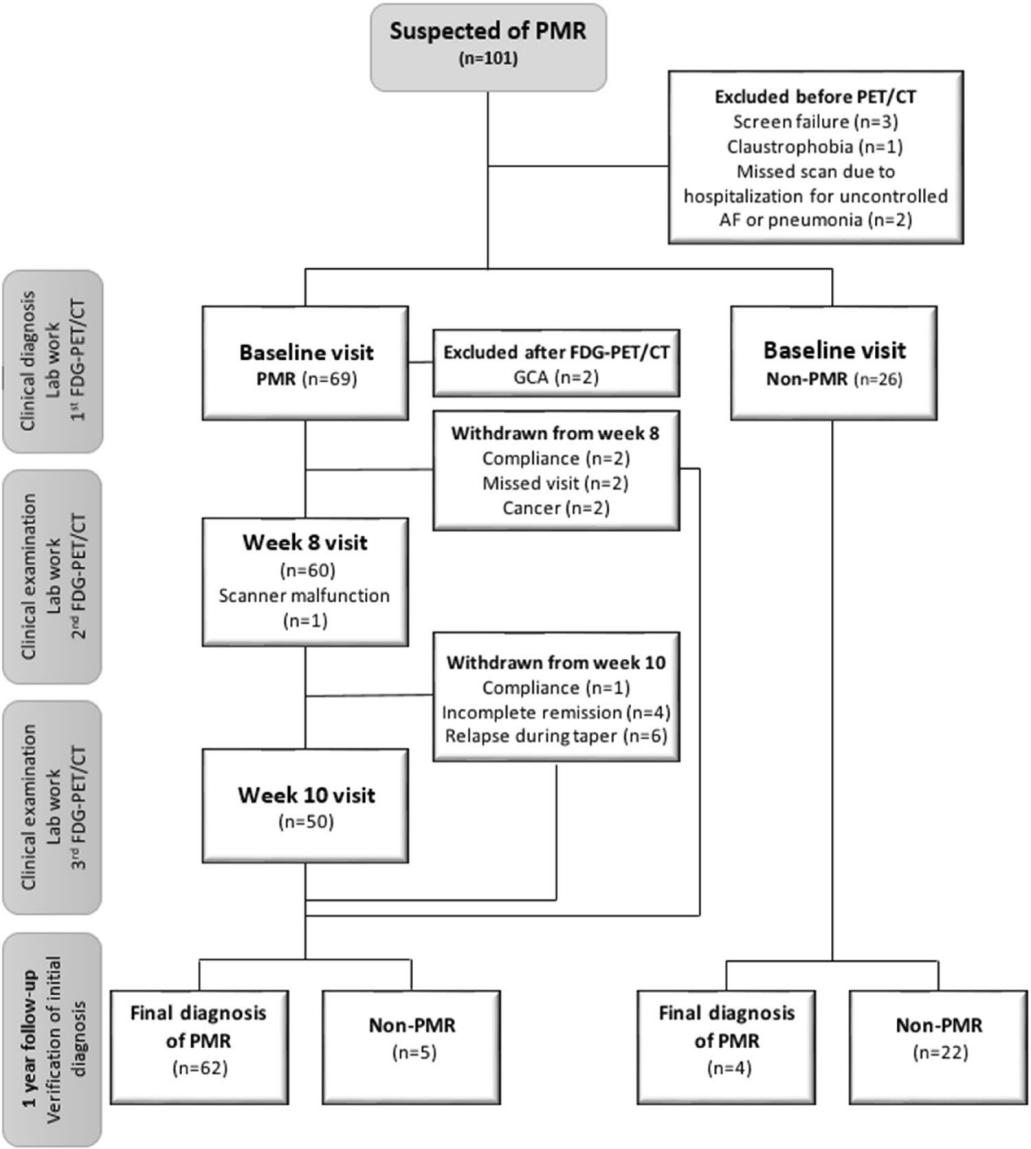
Flow chart of study flow. AF, Atrial fibrillation; PMR, polymyalgia *rheumatica*; GCA, giant cell arteritis; FDG-PET/CT, 2-[18F]fluoro-2-deoxy-D–glucose positron emission tomography/computed tomography

After 1 year of follow-up, a final diagnosis of isolated PMR was established in 68 patients, while 27 patients were diagnosed with another condition (Fig. 1). The 1-year diagnoses of the 27 non-PMR patients were as follows: reactive inflammatory conditions (*n* = 8), osteoarthritis (*n* = 8), rheumatoid arthritis (*n* = 3), paraneoplastic syndrome (*n* = 2), chronic pain condition (*n* = 2), peripheral artery disease (*n* = 1), adverse event to medication (*n* = 1), hyperthyroidism (*n* = 1), and unspecific hip pain (*n* = 1). Patients experiencing reactive inflammatory conditions presented with PMR mimicking symptoms for durations ranging from 2 to 28 weeks (median 11 weeks). These conditions included PMR-like symptoms following COVID-19 vaccine treated with NSAID (*n* = 1); symptoms emerging after diverticulitis and urinary tract infection resolving without treatment (*n* = 1); mild PMR symptoms along with elevated CRP, which resolved spontaneously without a history of infection or vaccine (*n* = 2); symptoms following COVID-19 infection treated with steroid injections and later with methotrexate and adalimumab (*n* = 1); and asymmetrical arthritis managed with prednisolone (*n* = 1). Furthermore, two patients were initially diagnosed with PMR and were treated with prednisolone for 8 weeks (*n* = 2), after which the medication was discontinued without relapsing symptoms. Subsequent evaluation regarded the symptoms as reactive, associated to a urinary infection and a COVID-19 vaccine (Supplementary Figure S2). In general, the patients with a confirmed clinical diagnosis of PMR after 1 year were more likely to have bilateral shoulder pain, restricted range of shoulder motion, and experience shoulder joint tenderness at baseline examination (Table 1). Compared to non-PMR, the group diagnosed with PMR had significantly higher baseline levels of CRP, global physician VAS score, and patient-reported pain VAS score. Consequently, the PMR-AS was significantly different between the groups. Moreover, individuals with a 1-year clinical diagnosis of PMR were more likely to have a clinical baseline diagnosis of PMR with a sensitivity/specificity of 93%/82% as well as meeting the clinical ACR/EULAR classification criteria at baseline, demonstrating a sensitivity/specificity of 79%/70% for the PMR diagnosis (Table 2).

**Table 1:** Baseline characteristics of patients with PMR, all non-PMR, and non-PMR with other inflammatory diseases

	PMR, *n* = 66^	Non-PMR, *n* = 27	*p*-value	Inflammatory conditions, *n* = 13	*p*-value
Age, mean ± SD	72 ± 7.1	69 ± 9.1	***p* < 0.05**	70 ± 9.4	*p* = 0.25
Sex (female), *n* (%)	27 (41%)	13 (48%)	*p* = 0.66	4 (31%)	*p* = 0.49
Disease duration in weeks, median (IQR)	8 (4–12)	14 (7–32)	***p* < 0.01**	9 (4–12)	*p* = 0.91
Symptoms					
Bilateral shoulder pain, *n* (%)	62 (94%)	18 (67%)	***p* < 0.01**	9 (69%)	***p* < 0.05**
Bilateral hip girdle pain, *n* (%)	56 (85%)	18 (67%)	***p* < 0.05**	9 (69%)	*p* = 0.23
Morning stiffness > 45 min, *n* (%)	44 (67%)	12 (44%)	***p* < 0.05**	6 (46%)	*p* = 0.21
Peripheral joint pain (other than shoulder and hips), *n* (%)	29 (44%)	15 (56%)	*p* = 0.31	8 (62%)	*p* = 0.25
Weight loss > 2 kg, *n* (%)	12 (18%)	4 (15%)	*p* = 0.77	2 (15%)	*p* = 1.0
Examination					
Shoulder—restricted range of motion, *n* (%)	49 (74%)	5 (19%)	***p* < 0.001**	4 (31%)	***P* < 0.01**
Shoulder joint tenderness, *n* (%)	46 (70%)	9 (33%)	***p* < 0.01**	3 (23%)	***p* < 0.01**
Hip—restricted range of motion, *n* (%)	36 (55%)	9 (33%)	*p* = 0.06	3 (23%)	***p* < 0.05**
Peripheral swollen joints (other than shoulder and hips), *n* (%)	9 (14%)	3 (11%)	*p* = 1.0	2 (15%)	*p* = 1.0
Comorbidities					
Hypertension, *n* (%)	32 (48%)	11 (41%)	*p* = 0.50	4 (31%)	*p* = 0.24
Hypercholesterolemia, *n* (%)	26 (39%)	4 (15%)	***p* < 0.05**	1 (8%)	***p* < 0.05**
Diabetes, *n* (%)	6 (9%)	5 (19%)	*p* = 0.29	1 (8%)	*p* = 1.0
Osteoporosis, *n* (%)	6 (9%)	0 (0%)	*p* = 0.18	0 (0%)	*p* = 0.58
Lab work					
CRP (mg/L), median (IQR)	35 (22–54)	10 (4–35)	***p* < 0 .001**	29 (16–92)	*p* = 0.60
ESR (mm)*, mean ± SD	45 ± 22	31 ± 20	***P* < 0.05**	36 ± 17	*p* = 0.19
RF > 5 kIU/L, *n* (%)**	7 (11%)	5 (19%)	*p* = 0.31	2 (15%)	*p* = 0.64
ACPA > 10 kIU/L, *n* (%)**	0 (0%)	1 (4%)	*p* = 0.28	1 (8%)	*p* = 0.17
PMR activity					
Treating physician global VAS score, median (IQR)	6 (5–7)	2 (1–4)	***p* < 0.001**	4 (2–6)	***p* < 0.05**
Patient-reported pain VAS score, median (IQR)	7 (6–8)	5 (5–7)	***p* < 0.01**	7 (6–8)	*p* = 0.30
PMR activity score^, median (IQR)	26 (20–33)	14 (7–23)	***p* < 0.001**	21 (16–24)	*p* = 0.06

Comparisons between the PMR and non-PMR group, as well as between the PMR and the non-PMR subgroup with inflammatory diseases, are presented with two-tailed *p*-values. ^Two patients who initially received a baseline diagnosis of giant cell arteritis were excluded from the analysis. *Missing data of 7 PMR patients and 2 non-PMR patients. **Missing data of 1 non-PMR patient. *PMR* Polymyalgia rheumatica, *SD* standard deviation, *IQR* interquartile range, *CRP* C-reactive protein, *ESR* erythrocyte sedimentation rate, *RF* rheumatoid factor, *ACPA* anti-citrullinated protein antibodies

**Table 2:** Diagnostic accuracy at baseline of FDG-PET/CT scoring methods, ACR/EULAR classification criteria, and a clinical diagnosis

	PMR group (*n* = 66)^	Non-PMR (*n* = 27)	Inflammatory conditions (*n* = 13)*
FDG-PET/CT—Leuven score			
Positive PET of PMR, *n* (%)	57 (86%)	10 (37%)	8 (62%)
Sensitivity (95% CI)		86.4 (75.7–93.6)	86.4 (75.7–93.6)
Specificity (95% CI)		63.0 (42.4–80.6)	38.5 (13.9–68.4)
FDG-PET/CT—dichotomous scoring			
Positive PET of PMR, *n* (%)	54 (82%)	8 (30%)	6 (46%)
Sensitivity (95% CI)		81.8 (70.4–90.2)	81.8 (70.4–90.2)
Specificity (95% CI)		70.4 (49.8–86.3)	53.9 (25.1–80.8)
ACR/EULAR classification criteria			
Positive clinical EULAR, *n* (%)	52 (79%)	8 (30%)	4 (31%)
Sensitivity (95% CI)		78.8 (67.0–87.9)	78.8 (67.0–87.9)
Specificity (95% CI)		70.4 (49.8–86.3)	69.2 (38.6–90.9)
Clinical baseline diagnosis			
Baseline diagnosis of PMR, *n* (%)	62 (94%)	5 (19%)	4 (31%)
Sensitivity (95% CI)		93.4 (85.2–98.3)	93.4 (85.2–98.3)
Specificity (95% CI)		81.5 (61.9–93.7)	69.2 (38.6–90.9)

The sensitivity and specificity for a baseline diagnosis of PMR are presented using both all non-PMR patients as controls, as well as the subgroup consisting of only non-PMR patients with inflammatory diseases. The 1-year clinical diagnosis serves as the reference standard. ^Two patients who initially received a baseline diagnosis of giant cell arteritis were excluded from the analysis. *Control patients with inflammatory diseases included reactive inflammatory conditions (*n* = 8), rheumatoid arthritis (*n* = 3), and paraneoplastic syndrome (*n* = 2). *PMR* polymyalgia rheumatic, *95% CI* 95% confidence interval, *FDG-PET/CT* 2-[18F]fluoro-2-deoxy-D-glucose–positron emission tomography/computed tomography

Among the included patients, the first FDG-PET/CT was performed a median of 3 days after the primary visit (range 1–12 days). The first FDG-PET/CT confirmed GCA in 2 patients, who were excluded from the analyses (Fig. 1). Excluding the GCA patients, complete remission was achieved on 15 mg prednisolone in all but 10 patients. Among these, 9 patients had their dosage escalated to 20–25 mg within the initial 4 weeks, while 1 patient continued with a 15 mg dosage. Over the course of the 1-year follow-up period, 5 patients (7%) who were initially clinically diagnosed with PMR had their initial diagnosis revised (Fig. 1, Supplementary Figure S2). Additionally, among the 26 patients initially clinically diagnosed with other medical conditions, 4 of them (15%) experienced a diagnostic shift to PMR following the 1-year follow-up period (Fig. 1, Supplementary Figure S3).

### FDG-PET/CT data

The Leuven score demonstrated a baseline sensitivity/specificity of 86%/63%, whereas the dichotomous score had a sensitivity/specificity of 82%/70% using the 1-year follow-up diagnosis as the reference standard (Table 2). A subgroup analysis in the non-PMR group showed that 8 out of 13 patients (62%) with inflammatory diseases (reactive inflammatory conditions, rheumatoid arthritis, and paraneoplastic syndrome) had a positive Leuven score, whereas this was only the case in 2 out of 14 patients with other conditions (14%) (*p* = 0.02). Applying the dichotomous score, 6 out of 13 patients (46%) with non-PMR inflammatory diseases were rated with a positive FDG-PET/CT compared to 1 out of 14 (7%) with other conditions (*p* = 0.03). In a subgroup analysis, comparing the group of inflammatory controls to the PMR group, the Leuven score showed a specificity of 39%, while the dichotomous score exhibited a specificity of 54% (Table 2). Further baseline characteristics of the inflammatory controls are presented in Table 1.

**Fig. 2:**
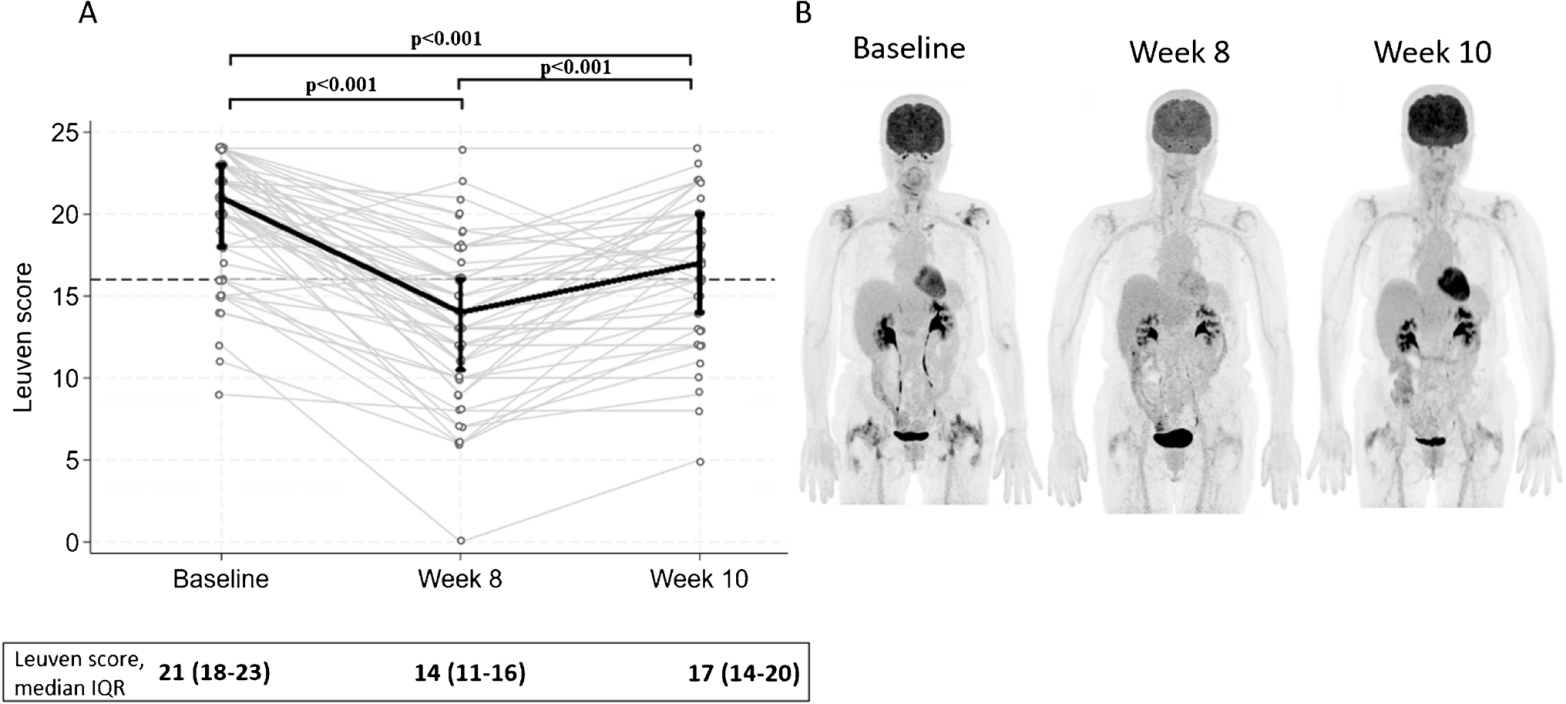
FDG-PET/CT Leuven score of patients with a 1-year diagnosis of PMR at diagnosis, after 8 weeks of prednisolone treatment, and during short-term discontinuation. **A** Scatter plot of the Leuven score at baseline, week 8, and week 10 for PMR patients. Black vertical lines marking interquartile ranges with connective lines between medians. Grey lines connecting observations between the three time points for each individual. Dashed line marks the Leuven cut-off value of 16. **B** PET scan of a PMR patient at the three time points

At week 8, all PMR patients had been tapered to 10–15 mg prednisolone daily and the Leuven score significantly decreased compared to baseline, whereas a short-term prednisolone discontinuation resulted in a significant increase at week 10 (Fig. 2). As a result, the sensitivity of a positive PET Leuven score/dichotomous score was reduced to 36%/41% at week 8 and subsequently improved to 66%/60% at week 10 (Table 3). The interrater reliability for the Leuven score showed the best agreement at baseline (ICC, 0.87 [CI 95%, 0.65–0.93]), the lowest during prednisolone treatment at week 8 (ICC, 0.66 [CI 95%, 0.13–0.85]), and a good level of agreement at week 10 (ICC, 0.79 [CI 95%, 0.36–0.91]).

**Table 3:** Clinical and FDG-PET/CT findings at baseline, after 8 weeks of prednisolone treatment, and during short-term discontinuation in patients with a 1-year diagnosis of PMR

	Baseline (*n* = 66)^		Week 8 (*n* = 56)*	Week 10 (*n* = 47)**
Age at baseline, mean ± SD	72 ± 7.1		72 ± 6.1	72 ± 6.5
Sex (female), *n* (%)	27 (41%)		23 (41%)	20 (43%)
Symptoms				
Bilateral shoulder pain, *n* (%)	62 (94%)		9 (16%)	18 (38%)
Bilateral hip girdle pain, *n* (%)	56 (85%)		6 (11%)	15 (32%)
Morning stiffness > 45 min, *n* (%)	44 (67%)		8 (14%)	13 (28%)
Peripheral joint pain (other than shoulder and hips), *n* (%)	29 (44%)		11 (20%)	11 (23%)
Examination				
Shoulder—restricted range of motion, *n* (%)	49 (74%)		4 (7%)	1 (2%)
Shoulder joint tenderness, *n* (%)	46 (70%)		9 (16%)	17 (36%)
Hip—restricted range of motion, *n* (%)	36 (55%)		7 (13%)	9 (19%)
Peripheral swollen joints (other than shoulder and hips), *n* (%)	9 (14%)		2 (4%)	4 (9%)
Lab work				
C-reactive protein (mg/L), median (IQR)	35 (22–54)		4 (4–8)	16 (5–32)
PMR activity				
Treating physician global VAS score, median (IQR)	6 (5–7)		1 (0–1)	1 (0–4)
Patient-reported pain VAS score, median (IQR)	7 (6–8)		1 (0–3)	3 (1–7)
PMR activity score, median (IQR)	26 (20–33)		2 (1–8)	7 (4–18)
FDG-PET/CT—Leuven score				
Leuven score, median (IQR)	21 (18–23)		14 (11–16)	17 (14–20)
Positive PET of PMR, *n* (%)	57 (86%)		20 (36%)	31 (66%)
Sensitivity (95% CI)	86.4 (75.7–93.6)	35.7 (23.4–49.6)		66.0 (50.7–79.1)
FDG-PET/CT—dichotomous scoring				
Positive PET of PMR, *n* (%)	54 (82%)		23 (41%)	28 (60%)
Sensitivity (95% CI)	81.8 (70.4–90.2)	41.1 (28.1–55.0)		59.6 (44.3–73.6)

^Two patients who initially received a baseline diagnosis of giant cell arteritis were excluded from the analysis. *Three patients whose diagnosis was revised to non-PMR at the 1-year visit and one patient who did not undergo FDG-PET/CT due to a scanner breakdown were excluded from the analysis. **Three patients whose diagnosis was revised to non-PMR at the 1-year visit were excluded from the analysis. *PMR* polymyalgia rheumatica, *SD* standard deviation, *IQR* interquartile range, *CRP* C-reactive protein, *FDG-PET/CT* 2-[18F]fluoro-2-deoxy-D-glucose–positron emission tomography/computed tomography

**Fig. 3:**
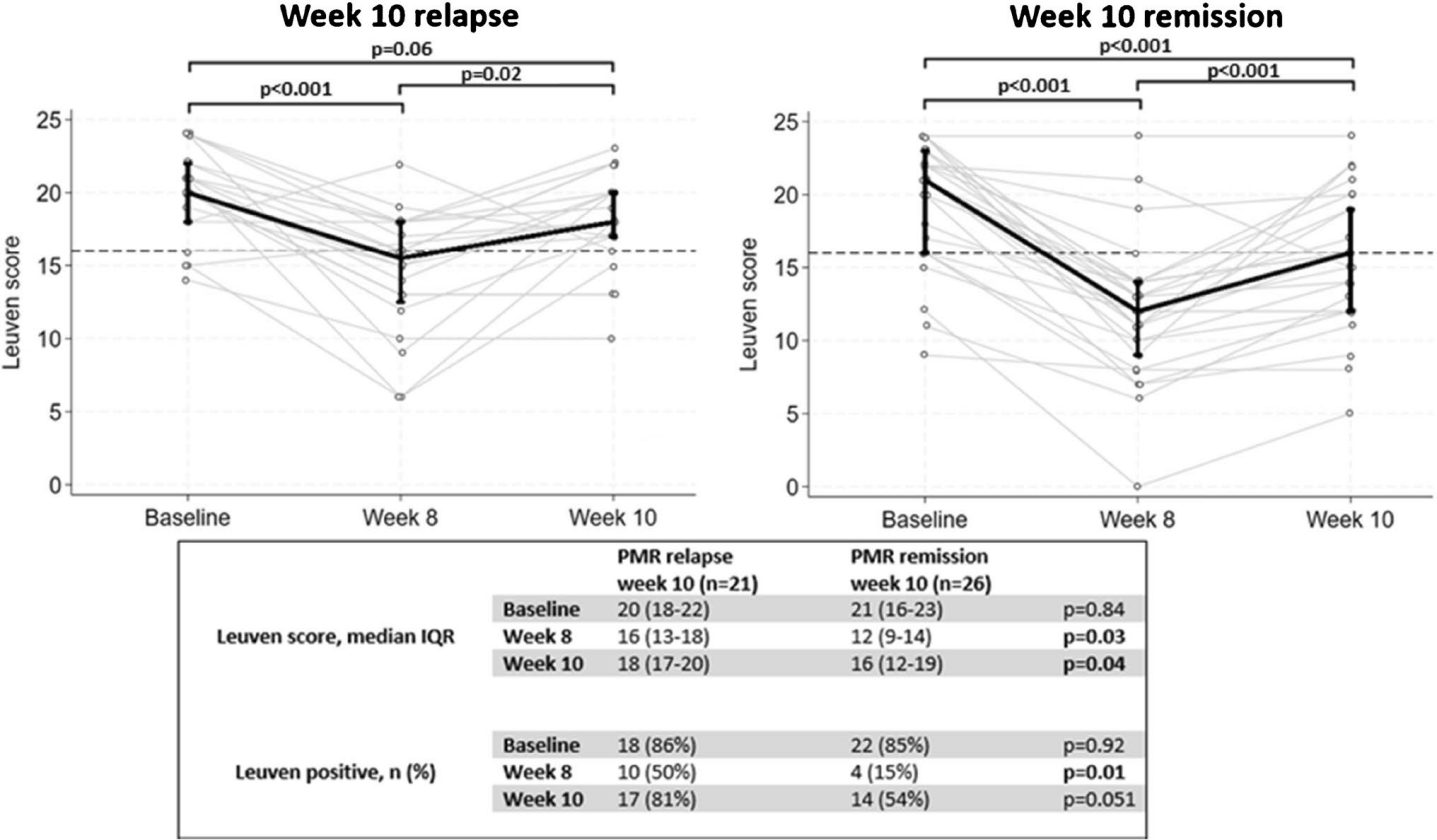
FDG-PET/CT Leuven score progression of patients with a 1 year-diagnosis of PMR divided into patients with relapse or remission after short-term prednisolone discontinuation. Scatterplot of the FDG-PET/CT Leuven score at baseline, week 8, and week 10 for PMR patients with relapse and remission at 10 weeks. Black vertical lines marking interquartile ranges with connective lines between medians. Grey lines connecting observations between the three time points for each individual. Dashed line marks cut-off value for Leuven score. The table under the scatter plots shows Leuven score and proportions of positive individuals. At 8 weeks, one relapsing individual missed the FDG-PET/CT (scanner breakdown). IQR, interquartile range

Upon subgrouping PMR patients at the 10-week visit into those experiencing relapse and those still in remission after steroid discontinuation, it was observed that the relapse group had a significantly higher proportion of Leuven positive individuals at week 8 (Fig. 3). Despite the divergence in PET results at week 8, both groups exhibited a low clinical disease activity including a PMR-AS below 7 (Supplementary Table S1). At week 10, the relapse patients exhibited more severe symptoms of PMR and had a tendency towards a higher proportion of Leuven positive individuals (*p* = 0.051) (Fig. 3). Comparing these two groups at baseline, the only significant difference observed was related to shoulder joint palpation tenderness (Supplementary Table S1).

## Discussion

This is the first study to demonstrate that FDG-PET/CT has limited capability for differentiating PMR from other inflammatory diseases. Furthermore, we provide pioneering data showing the effect of prednisolone for the diagnostic accuracy of FDG-PET/CT in PMR.

The sensitivities of the Leuven composite score and the dichotomous score resembled the previously reported sensitivities, ranging between 85 and 91% for the Leuven score, in prednisolone naïve patient cohorts of suspected PMR [11, 12, 25]. The dichotomous score was introduced to reflect the diagnostic procedure in a routine clinical setting, where the Leuven score may not be applied. This rationale also guided the decision not to incorporate maximal standard uptake value analyses in this study, as such analyses are not recommended for diagnostic purposes [32]. In our study, the specificity of the two scoring methods was remarkably lower compared with the previously reported specificities ranging between 84 and 98% for the Leuven score [11, 12, 25]. This can be explained by a greater proportion of non-PMR patients with other inflammatory diseases within this study (48% vs 19–32%), who exhibit a closer resemblance to patients with PMR. These patients displayed FDG-PET/CT scans that more frequently were positive compared to controls with other conditions. As a result, this subgroup of patients might be predisposed to overtreatment. This was exemplified by one patient with a reactive disease who presented with typical symptoms of PMR as well as a Leuven positive FDG-PET/CT (Supplementary Figure S1, C). The FDG-PET/CT images exhibited a less typical pattern of PMR with extensive uptake along muscle tendons, prompting deliberation within the nuclear physician team. However, following discussion, it was ultimately determined that this pattern bore a resemblance to PMR. Nonetheless, subsequent follow-up revealed the condition to be a reactive response to a COVID-19 vaccine [35]. Therefore, other inflammatory diseases may in general exhibit a less typical FDG-uptake pattern and scoring systems designed to capture this are warranted.

This study is the first to demonstrate the effect of prednisolone for the diagnostic sensitivity of FDG-PET/CT in PMR. A controlled reduction in prednisolone dose, followed by a short-term discontinuation, significantly enhanced the diagnostic sensitivity of FDG-PET/CT for PMR. This underscores the importance of discontinuing prednisolone in patients suspected of PMR before undergoing a confirmative FDG-PET/CT scan, particularly considering that prednisolone treatment for 8 weeks approximately halved the diagnostic sensitivity and reduced the interrater reliability. Previous studies have implied that the diagnostic accuracy of FDG-PET/CT for PMR diminishes with the administration of glucocorticoids. However, none of these studies was explicitly designed to investigate the direct impact of prednisolone on diagnostic accuracy or whether prednisolone should be tapered prior to the FDG-PET/CT [19, 23, 24, 36, 37]. In the study by Brinth et al. [23], a retrospective comparative analysis was conducted including individuals suspected of PMR as well as individuals with other diseases. The results revealed a sensitivity of 81% for the Leuven score among patients who had not been exposed to prednisolone, as opposed to a 24% sensitivity in the group undergoing glucocorticoid treatment. A few studies have reported similar retrospective data from cohorts of PMR patients, demonstrating elevated FDG uptake and a higher number of sites with FDG uptake in steroid-naive patients [19, 24, 37]. Blockmans et al. [36] have conducted a prospective study with repeated PET scans in a cohort of confirmed PMR patients. The data of 22 patients demonstrated a reduction in FDG uptake around the shoulder, hips, and processi spinosi following 3 months of prednisolone treatment. However, this study did not investigate diagnostic accuracy and the effect of prednisolone discontinuation. Given that many patients will already have commenced glucocorticoid treatment prior to FDG-PET/CT referral, our study suggests to taper prednisolone to 5 mg for 1 week, followed by a 1-week discontinuation, before performing FDG-PET/CT scans to increase the diagnostic accuracy and improve interrater consistency. However, it is essential to acknowledge that this suggestion is based on study participants receiving prednisolone for precisely 8 weeks before the short-term taper and discontinuation.

The subgroup analysis, comparing patients in remission after 10 weeks against those with relapse symptoms, revealed that the FDG-PET/CT could not distinguish between these two groups at baseline. However, at 8 weeks, the relapse group exhibited a higher Leuven score, resulting in a higher proportion of individuals with a positive Leuven score. This phenomenon was present despite the fact that CRP levels remained within the normal range for both groups, and both cohorts displayed a low disease activity as indicated by a PMR-AS score of less than 7. Overall, our results imply that FDG-PET/CT signs of PMR may be present prior to the onset of symptoms and elevation of the CRP level in patients with PMR.

PMR patients with relapse at week 10 exhibited clinical and biochemical signs indicative of relapse, in contrast to the remission group. Furthermore, the relapse group showed a tendency towards a higher proportion of Leuven positive individuals compared to the remission group, but this difference was not statistically significant. This result may be underestimated since 10 patients, who experienced incomplete remission or relapse, were unable to complete the short-term prednisolone discontinuation. Nevertheless, it might indicate that patients should ideally undergo prednisolone cessation until they exhibit relapse symptoms to improve the diagnostic accuracy of a subsequent FDG-PET/CT. However, larger studies are needed to confirm this statement.

There are limitations in this study. Similarly, to all previous studies, the clinicians responsible for determining the final PMR diagnosis after 1 year were not blinded for the FDG-PET/CT results due to legal and ethical considerations. However, in this study, the clinical PMR diagnosis was intentionally established prior to the conduction of FDG-PET/CT scans. Among the patients initially diagnosed with PMR, no alterations in diagnosis occurred during the initial 8-week period of prednisolone treatment, except for two patients who were subsequently diagnosed with cancer and two patients with coexisting GCA. Consequently, the final diagnosis rested predominantly on the clinical assessment conducted prior to the FDG-PET/CT, coupled with the evaluation of the response to prednisolone treatment. Another general limitation shared with all previous studies is the absence of systematic documentation regarding pre-screening exclusions before clinical assessment. Consequently, we cannot completely eliminate the risk of a selection bias within this cohort. However, the implementation of rapid referral clinics ensured the inclusion of a unique cohort that represents the entirety of the PMR population. The general practitioners were actively encouraged to refer all suspected PMR individuals to our rapid access clinics. This ensured that the composition of the control group closely resembled what has been previously reported in studies evaluating the rapid access strategy [38]. Thus, we believe that the extent of selection bias typically encountered in hospital-based cohorts might not have been as pronounced in this investigation compared to studies that apply the conventional referral procedure, where only approximately 25% of the entire PMR population is referred from primary care [14]. Finally, in this study, we opted to evaluate the FDG-PET/CT images using only the Leuven score as well as a dichotomous score. Although other less validated scoring methods have been developed, these mainly rely on retrospective data or have selected control groups not necessarily comprising suspected PMR patients [12, 19–24]. As a result, we considered the Leuven score to be the most reliable scoring method.

## Conclusion

This is the first prospective study performing FDG-PET/CT in treatment naïve patients suspected of PMR and including the effect of prednisolone treatment and a short-term discontinuation. The study shows that FDG-PET/CT has limited diagnostic accuracy for differentiating PMR from other inflammatory diseases. Furthermore, we provide pioneering data showing that prednisolone treatment considerably reduces the diagnostic sensitivity of FDG-PET/CT for PMR, whereas a short-term discontinuation can increase the sensitivity, although it remains below the pre-treatment level.

### Supplementary information

Below is the link to the electronic supplementary material.

Supplementary file1 (DOCX 1274 KB)

## Data Availability

Data are available on reasonable request.
