# Supplementary material for: The effect of prednisolone and a short-term prednisolone discontinuation for the diagnostic accuracy of FDG-PET/CT in polymyalgia rheumatica—a prospective study of 101 patients
Source: Eur J Nucl Med Mol Imaging. 2024 Apr 2;51(9):2614–24. doi: 10.1007/s00259-024-06697-8 (PMC11224098; doi:10.1007/s00259-024-06697-8)
Supplement: Supplementary file 1 — Supplementary file1 (DOCX 1274 KB) [file 259_2024_6697_MOESM1_ESM.docx]

Journal: European Journal of Nuclear Medicine and Molecular Imaging

**The effect of Prednisolone and a Short-term Prednisolone Discontinuation for the Diagnostic Accuracy of FDG-PET/CT in Polymyalgia Rheumatica – a Prospective Study of 101 Patients**

Andreas Wiggers Nielsen^1,2,3^, Ib Tønder Hansen^1,2^, Berit Dalsgaard Nielsen^1,2,4^, Søren Geill Kjær^3^, Jesper Blegvad-Nissen^3^, Kate Rewers^5^, Christian Møller Sørensen^4^, Ellen-Margrethe Hauge^1,2^, Lars Christian Gormsen ^2,6^, Kresten Krarup Keller ^1,2^

1. Department of Rheumatology, Aarhus University Hospital, Aarhus, Denmark
2. Department of Clinical Medicine, Aarhus University, Aarhus, Denmark
3. Diagnostic Centre, Silkeborg Regional Hospital, Silkeborg, Denmark
4. Department of Internal Medicine, Horsens Regional Hospital, Horsens, Denmark
5. Department of Nuclear Medicine and PET, Odense University Hospital, Odense, Denmark
6. Department of Nuclear Medicine and PET, Aarhus University Hospital, Aarhus, Denmark

**Corresponding author:** Andreas Wiggers Nielsen, Led- og Bindevævssygdomme, Palle Juul-Jensens Boulevard 59, 8200 Aarhus, Denmark. Mail: andrenie@rm.dk. Orchid ID: <https://orcid.org/0000-0001-8180-6871>


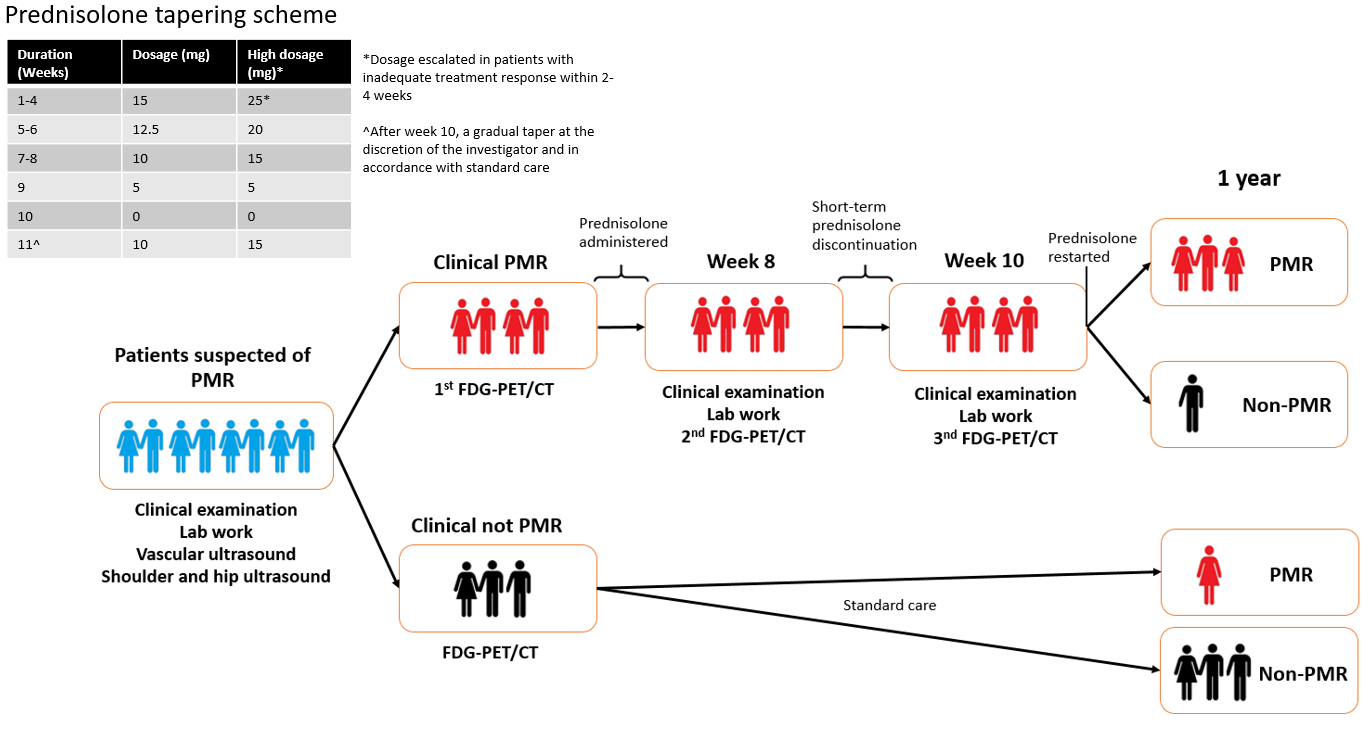


Supplementary Figure S1: Study design with weekly tapering scheme within the first 10 weeks.


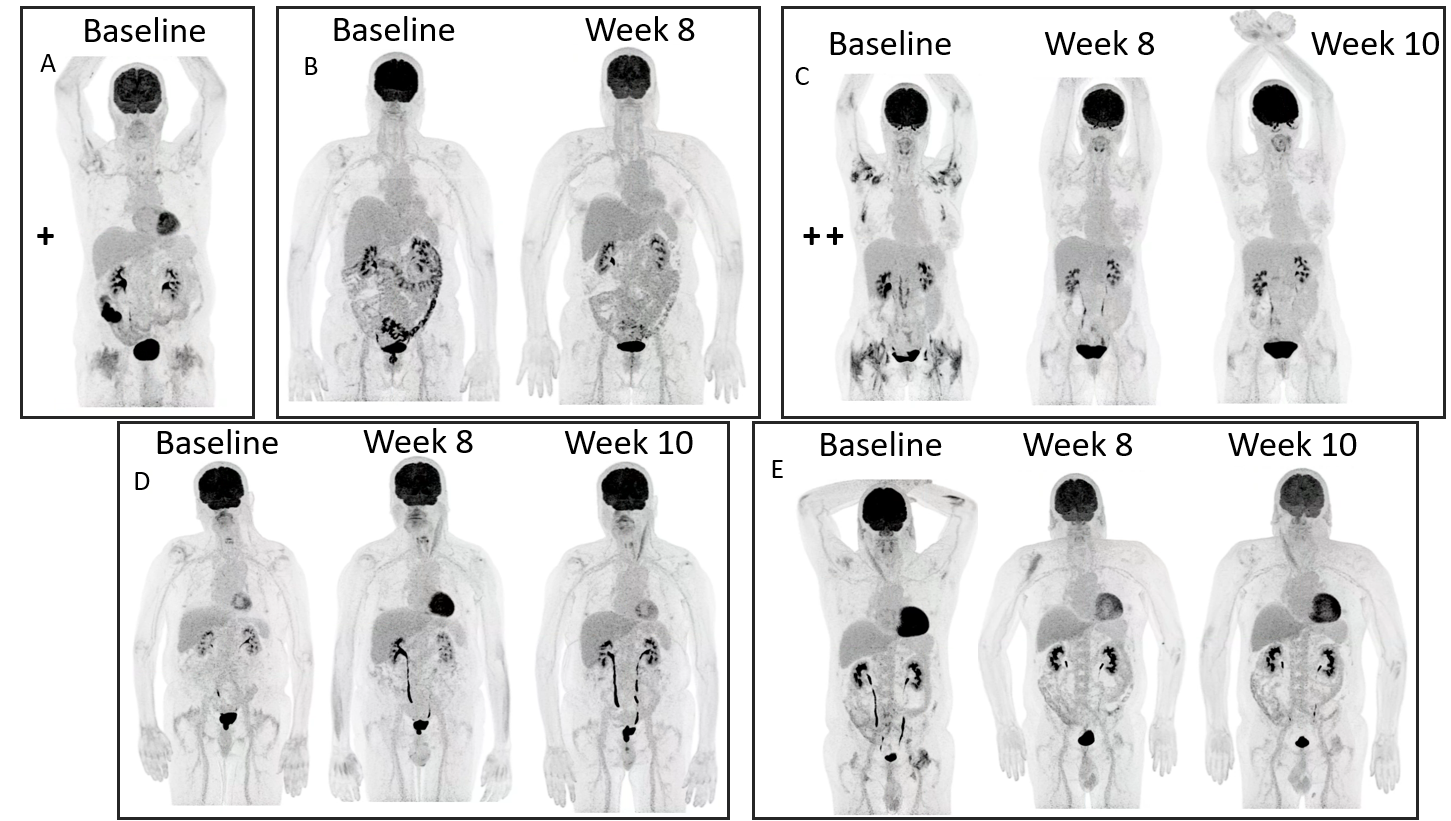


Supplementary Figure S2: PET/CT scans of patients with an initial clinical diagnosis of PMR subsequently changed to other conditions

A: Colon cancer depicted on the baseline PET/CT. Symptoms mimicking PMR at baseline, which resolved after tumor removal and without prednisolone administration. Consequently, the symptoms was attributed to a **paraneoplastic syndrome**. B: Patient presenting with bilateral girdle pain of the shoulder and hips as well as elevated CRP. The patient did not respond to prednisolone treatment. However, the symptoms resolved after discontinuation of thiazides and the initial symptoms were interpreted as **drug-induced myalgia.** C and D: Case C has previously been described [1]. Both patients presented with bilateral girdle pain of the shoulder and hips. Initially diagnosed as PMR, but the patients did not experience any symptoms during the short-term prednisolone cessation. Considering the clinical course and the possibility of an underlying **reactive disease** following a recent Covid-19 vaccine for patient C and urinary tract infection in patient D, the decision was made to refrain from restarting prednisone. Through the remaining follow-up period, both patients remained asymptomatic, leading to the conclusion that the initially symptoms could be attributed to **reactive diseases**. E: Patient presenting with bilateral girdle pain of the shoulder and hips as well as elevated CRP. At baseline, there was no signs of peripheral arthritis observed. However, during follow-up the patient developed synovitis in multiple finger joints, which aligned with the characteristics of **rheumatoid arthritis** As a result, the initial diagnosis of PMR was revised. +: The PET image was rated as Leuven positive, with a Leuven score of 16 or above. ++: The PET image was rated as positive for PMR both utilizing Leuven and the dichotomous score.


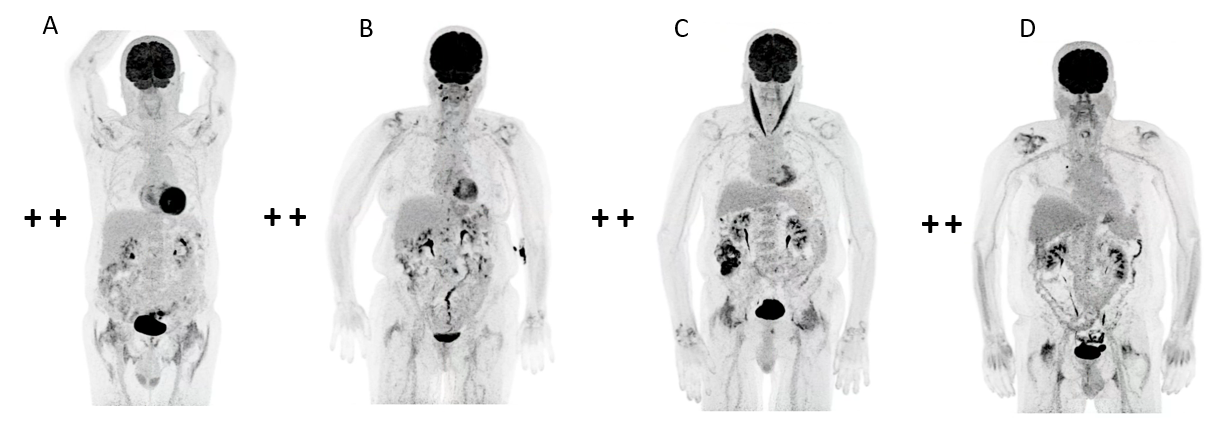


Supplementary Figure S3: Baseline scans of patients initial diagnosed with other conditions subsequently changed to PMR

A: Patient presenting with bilateral hip and shoulder girdle pain, along with pain and tenderness of multiple finger joints following a bowel infection. Initially, the symptoms were attributed to an **unspecific arthritis**. As the disease progressed, the symptoms related to the finger abated, while the hip and shoulder symptoms persisted. Consequently, prednisolone was administered with a favorable effect, prompting the diagnosis of PMR. B: Patient with an inflammatory bowel disease presenting with predominately neck and low back pain. Initially, these symptoms were ascribed to a **reactive disease** linked to bowel disease activity. After 6 weeks, the patient developed classic symptoms of PMR with bilateral shoulder and hip pain, morning stiffness and elevated CRP levels. As a result, the patient was initiated on prednisolone, with the swift resolution of the symptoms. C: Patient presenting with bilateral hip pain and morning stiffness, with the absence of shoulder ultrasound signs and symptoms. Initially diagnosed as a **reactive disease**. After 4 weeks, the patient returned exhibiting pain, morning stiffness and subdeltoid bursitis of both shoulders. Subsequently, the diagnosis was revised to PMR, and the patient displayed a rapid response to prednisolone. D: Patient presenting with hip and shoulder girdle pain, without ultrasound findings of bursitis, tendinitis or synovitis along with CRP levels in the normal range. Initially the symptoms were attributed to **intermittent claudication**. However, since the symptoms persisted with an increasing severity over time prednisolone was administered. This resolved the symptoms of the patient within hours, prompting the diagnosis of PMR. ++: The PET image was rated as positive for PMR both utilizing Leuven and the dichotomous score.

[1] Nielsen AW, Hansen IT, Gormsen LC, Hauge EM, Keller KK. Self-limiting reactive disease mimicking polymyalgia rheumatica following Moderna COVID-19 vaccine. Scand J Rheumatol 2022;51(5):411-3.

|  |  | Relapse week 10 (n=21) | PMR remission (n=26) | p-value |
| --- | --- | --- | --- | --- |
|  |  |  |  |  |
| C-reactive protein (mg/L), median (IQR) | **Baseline** | 32 (23-39) | 33 (15-48) | p=0.78 |
|  | **Week 8** | 4 (4-9) | 4 (4-6) | p=0.13 |
|  | **Week 10** | 20 (14-32) | 10 (4-22) | p=0.14 |
|  |  |  |  |  |
| Morning stiffness >45 min, n (%) | **Baseline** | 14 (67%) | 17 (65%) | p=0.93 |
|  | **Week 8** | 3 (14%) | 2 (8%) | p=0.64 |
|  | **Week 10** | 10 (48%) | 3 (12%) | **p<0.01** |
|  |  |  |  |  |
| Shoulder – Restricted range of motion, n (%) | **Baseline** | 17 (81%) | 18 (69%) | p=0.36 |
|  | **Week 8** | 1 (5%) | 0 (0%) | p=0.45 |
|  | **Week 10** | 9 (43%) | 2 (8%) | **p<0.01** |
|  |  |  |  |  |
| Shoulder joint palpation tenderness, n (%) | **Baseline** | 19 (90%) | 12 (46%) | **p<0.01** |
|  | **Week 8** | 5 (24%) | 0 (0%) | **p<0.05** |
|  | **Week 10** | 15 (71%) | 2 (8%) | **p<0.001** |
|  |  |  |  |  |
| Hip - Restricted range of motion, n (%) | **Baseline** | 10 (48%) | 14 (54%) | p=0.67 |
|  | **Week 8** | 1 (5%) | 4 (15%) | p=0.36 |
|  | **Week 10** | 6 (29%) | 3 (12%) | p=0.26 |
|  |  |  |  |  |
| Treating physician global VAS-score, mean ±SD | **Baseline** | 7 (5-7) | 5 (4-7) | p=0.32 |
|  | **Week 8** | 1 (0-1) | 0 (0-0) | **p<0.001** |
|  | **Week 10** | 4 (3-6) | 1 (0-1) | **p<0.001** |
|  |  |  |  |  |
| Patient reported pain VAS-score, mean ±SD | **Baseline** | 7 (6-8) | 7 (6-8) | p=0.92 |
|  | **Week 8** | 2 (0-4) | 0 (0-1) | **p<0.01** |
|  | **Week 10** | 7 (2-9) | 2 (0-3) | **p<0.001** |
|  |  |  |  |  |
| PMR activity score, mean ±SD | **Baseline** | 22 (19-29) | 24 (18-30) | p=0.84 |
|  | **Week 8** | 4 (1-8) | 1 (0-2) | **p<0.01** |
|  | **Week 10** | 13 (9-42) | 5 (3-7) | **p<0.001** |

**Supplementary Table S1 – Clinical characteristics at baseline, week 8, and week 10 of the PMR patients with relapse or remission at week 10**

At 8 weeks, one individual in the relapse group missed a PET/CT due to scanner breakdown. PMR: Polymyalgia rheumatica, IQR: interquartile range.
